# Preparing for export? Medical and nursing student migration intentions post-qualification in South Africa

**DOI:** 10.4102/phcfm.v5i1.483

**Published:** 2013-05-13

**Authors:** Gavin George, Candice Reardon

**Affiliations:** 1 Health Economics and HIV and AIDS Research Division, University of KwaZulu-Natal, South Africa

## Abstract

**Background:**

The migration of health professionals can have a profound impact on health systems around the globe. The International Organization for Migration's (IOM) Mobility of Health Professionals Research Project (MoHProf) aimed to improve knowledge about the migration of healthcare professionals and understand migration intentions and factors influencing the migration of medical and nursing students.

**Objectives:**

The study aimed to determine the proportion of nursing and medical students who were intending to emigrate, their attitudes and beliefs about, and the factors influencing their decision to emigrate.

**Method:**

This study was conducted at three medical schools and one nursing school in South Africa (*n* = 298) amongst 260 medical and 38 nursing students. One hundred and twenty-five students were in the final year of their studies and 143 were in their prefinal year. Thirty students did not indicate the year of their studies. Every student present on the day of data collection completed a questionnaire comprising psychometric and survey-based questions. Descriptive and inferential statistics were used to analyse the data.

**Results:**

More than a third (37%) of the respondents intended to work or specialise abroad. The majority of medical (58.9%) and nursing (66.6%) students intended to leave SA within five years of completing their medical or nursing studies. The perception of poor working conditions within the health sector, such as long work hours, high patient loads, inadequate resources and occupational hazards, influenced medical students to consider emigrating from South Africa.

**Conclusion:**

The high number of medical and nursing students intending to emigrate requires a reassessment of effectiveness of retention strategies for doctors and nurses in the South African healthcare system and actions to improve working conditions in the public healthcare sector.

## Abstrait

**Préparation à l'exportation? Médical et infirmier étudiant migration intentions qualification du message en Afrique du Sud**

**Contexte:**

La migration des professionnels de la santé peuvent avoir des répercussions profondes sur les systèmes de santé partout dans le monde. La mobilité des professionnels de la santé la recherche (projet MoHProf) visant à améliorer la connaissance de la migration des professionnels de la santé et de comprendre la migration intentions et les facteurs influant sur la migration des médecins et des étudiants en sciences infirmières.

**Objectifs:**

L’étude visait à déterminer la proportion des soins infirmiers et étudiants en médecine qui ont l'intention d’émigrer à l’étranger, leurs attitudes et leurs croyances, et les facteurs qui influent sur les élèves» décisions à émigrer.

**Méthodes:**

Cette étude a été menée dans trois écoles de médecine et une école d'infirmières en Afrique du Sud (SA) (*n* = 298). Les élèves ont rempli un questionnaire composé de qualités psychométriques et basé sur les données d'enquête et aux questions. Descriptif et inférence statistique ont été utilisés pour analyser les données.

**Résultats:**

Un tiers des personnes interrogées ont l'intention de travailler ou se spécialiser à l’étranger. La majorité destiné à laisser SA dans les cinq premières années de leur carrière médicale. Les résultats suggèrent que ces répondants étaient probablement l'intention d'attendre quelques années après l'obtention de leur diplôme, peut-être, afin de se spécialiser dans SA, enregistrer de l'argent pour les frais de l’émigration, ou pour terminer leur travail obligatoire engagements.

**Conclusions:**

Le montant substantiel des soins médicaux et infirmiers les étudiants désireux d’émigrer à l’étranger exige une réévaluation de l'efficacité des efforts de conservation pour les travailleurs de la santé dans la SA système de soins de santé et des mesures décisives pour améliorer les conditions de travail dans le secteur de la santé publique.

## Introduction

Since the turn of the century the impact of globalisation and increasingly porous country borders has facilitated an increase in the migration of healthcare practitioners (HP) between countries. This has resulted in a ‘reverse subsidy’ of HPs from poor to rich countries, as knowledge and skills are transferred from developing to developed nations. The unfortunate costs borne by host countries, in most cases developing countries, include weakened health systems, the erosion of health gains, the loss of intellectual capital and a diminishing return on the investment made by the country when it subsidised the education and training of these workers.^1^

In terms of actual migration flows, work permit data and registration data for 2001 and 2002 show there has been significant growth in the inflow of doctors to the United Kingdom. In 2002, nearly half of all the new full registrants on the UK General Medical Council (GMC) register were foreign,^2^ and of those, 3509 were South African trained doctors. In the same year 1950 South African doctors were reportedly living in the USA.^1^ In 2002, 2884 South African nurses were documented to have emigrated from South Africa (SA) to the UK,^3^ the number had decreased to 1418 in 2003.^4^ By 2004, 7% of the total nursing workforce in SA had left SA to work in seven Economic Co-operation and Development (OECD) countries (Denmark, Canada, Finland, Portugal, USA, UK and Ireland), whilst slightly over a third of the South African doctors (37%, *n* = 12 136) who had been trained in SA were living in eight OECD member countries (Australia, Canada, Finland, France, Germany, Portugal, USA and UK).^3^ Moreover, by 2006 the proportion of South African doctors who had left the country rose to 40%.^1^ Statistics from the South African Department of Health indicate that in 2006 almost 9000 SA doctors (*n* = 8921) and 6844 nurses and midwives were working in Australia, Canada, New Zealand, UK and USA.^5^

Research has found^6, 7^ a high degree of emigration intention amongst health science students in southern Africa, with 65% indicating that they would like to emigrate within five years of graduating. Health science students in the study by Crush, Pendleton and Tevera^6^ were noted to have given more thought to moving to another country and to be inclined to stay in their destination country for a longer period of time than non-health science students. Although lower than the proportion found by Crush et al.,^6^ a study amongst 194 medical students in the province of KwaZulu-Natal showed that roughly one in five students intended to practise abroad after graduation, whilst fewer wished to pursue postgraduate training abroad (*n* = 10, 7.6%)^7^. Emigration considerations were also prevalent amongst a sample of 9743 students from sub-Saharan Africa which included 4532 SA students. Approximately four in five students (79%) had thought about moving to another country, and 35% said that they would consider the possibility during the next six months. The proportion of final year students in the study, however, who had actually taken active steps to leave the country was much lower; only 19% had applied or were in the process of applying for work permits abroad, whilst 11% were applying for permanent residence or citizenship elsewhere.^6^

Factors influencing individuals’ decisions to migrate to other countries are commonly grouped into push and pull factors depending on whether the factor is located in the source or destination country. Prominent push factors for HPs in sub-Saharan Africa include resource-limited health care systems, deteriorating work environments, human resource shortages, low salaries, political tensions and upheaval, gender discrimination, lack of personal security, HIV and AIDS, and deteriorating quality of life and social systems such as education and welfare.^1, 9, 10^ Salient pull factors attracting HPs to developed countries abroad often relate to the availability of jobs in the destination country, more manageable workloads, higher remuneration, better working conditions, safer living environments, higher quality of life and a more economically and politically stable country.^1, 9, 10^ Although few studies have explicitly set out to determine the variation of push and pull factors across age groups, it is interesting to note that Maslin^11^ found that HPs between the ages of 20 and 29 were more likely to cite deteriorating working conditions as a reason for leaving SA than their older counterparts.

Research amongst South African HPs revealed that financial factors, better job opportunities for themselves and better schooling opportunities for their children abroad as well as the high crime rate in SA were significant factors encouraging these health workers to emigrate.^12^ Further, the opportunity to gain international experience is an important pull factor attracting South African HPs, particularly doctors, to overseas countries.^11^ According to Oberoi and Lin,^10^ however, push factors motivating HPs to leave SA can exert a more powerful influence on their emigration decisions than the factors attracting them to their destination country. The major push factors that emerged most frequently in their interviews with South African doctors who had emigrated to Australia were poor remuneration, lack of job satisfaction, lack of future prospects for further education and career development, poor working conditions, HIV and AIDS and poor quality of life.^10^

### Objective

The study examined medical and nursing students’ intentions and reasons for wanting to emigrate after graduation to gain a sense of the prevalence of those desires and of the factors that influenced their decisions. There appears to be few studies in sub-Saharan Africa that have investigated students’ attitudes and beliefs about emigration and the association these have with their intentions to leave the country. Push and pull factors influencing decisions to emigrate have been better documented, although most of this research has been conducted amongst qualified HPs with fewer studies conducted amongst current medical and nursing students.^6, 7^

Hence, this study aimed to achieve three objectives:

1. To determine the proportion of nursing and medical student respondents who intend to emigrate within five years of graduation.
2. To assess the attitudes and beliefs of respondents intending to emigrate.
3. To examine the factors associated with respondents’ intentions to emigrate from SA.

### Contribution to the field

Evidence from previous studies indicates that a high proportion of nurses and doctors plan to leave or have already left the country. Knowledge about the migration intentions, attitudes and beliefs of medical and nursing students along with the factors influencing their migration, is urgently needed in order to develop policies to stem the flow of HPs from SA.

## Ethical Considerations

Ethical approval was gained from the University of KwaZulu-Natal (UKZN) Biomedical Research Ethics Committee (Ref: BE191/010) as well as from the KwaZulu-Natal provincial Department of Health (Ref: HRKM146/10). Approval to conduct the research study was also obtained from relevant gatekeepers in the three medical schools and one nursing school. All respondents involved in the study provided individual consent to participate. Electronic and hard copies of the data are stored safely at the Health Economics and HIV and Aids Research Division (HEARD) of University of KwaZulu-Natal offices, and are only accessible to the researchers involved in the study who are bound by confidentiality agreements. In this presentation of the findings, no identifying information of the respondents is provided in order to ensure the confidentiality of their accounts.

## Methods

### Design

A quantitative, cross-sectional design was used to examine the variables of interest within a large (*n* = 298) cohort of nursing and medical students.

### Sample Size

The study population comprised final and pre-final year nursing and medical students that were enrolled at three medical and one nursing school in SA. All eight medical schools in South Africa were approached for the study but due to the time period of data collection, October to November, five of the schools declined the researchers admission to their students. Two nursing schools in KwaZulu-Natal (where the researchers are based) were approached about the study, but only one nursing school granted access to the researchers. Prefinal and final year students were purposively selected for the study because it was felt that as they were approaching the end of their studies, they would be engaging with questions about emigration to a greater extent than younger students. Altogether 298 medical and nursing prefinal and final year students were purposively sampled. Every medical and nursing student present in their classes on the day of data collection consented to participate in the study. The sample comprised of 260 medical students and 38 nursing students. One hundred and twenty-five students were in the final year of their studies, whilst 143 were in the prefinal year. Thirty students did not indicate the year of study.

### Materials

A questionnaire was developed for the study that was informed by relevant literature and empirical research. Questions were divided into seven sections: 1) Demographic information of respondents, 2) Measures of intent to go abroad, 3) Migration potential, 4) Sources of information about working or training abroad, 5) Factors that influenced their decisions to emigrate, 6) Beliefs about emigration and 7) Attitudes about emigration.

Apart from three questions drawn from three studies, the demographic questions were mostly developed by the researchers.^6, 7, 8^ Several of the items regarding Section 4 (Sources of information about emigration) were taken from Crush, Pendleton and Tevera.^6^ The push factors listed in Section 5 are a compilation of factors drawn from several authors.^6, 7, 8, 13, 14^ Question items from previous studies^6, 8, 13^ as well as items developed by the authors were used to develop 13 Belief statements. Thirteen question items enquiring about respondents’ attitudes to emigration were also developed by the researchers for the study. The alpha reliability for the Beliefs scale was originally .74. Two items were omitted because they were correlating negatively with other Belief items (‘The increase in salaries due to the OSD [Occupational Specific Dispensation] will ensure that more doctors and nurses stay in SA’ and ‘I will be exposed to fewer occupational risks abroad). After dropping these items the alpha coefficient increased slightly to 0.77. Initially the alpha reliability for the Attitudes scale was 0.58. The inter-item correlation matrix and item total statistics tables were inspected to see which items could be dropped to improve the internal consistency of the scale. Three items were dropped (‘Pursuing work opportunities abroad will give doctors and nurses valuable experience and skills they will not acquire in SA’, ‘The emigration decisions of doctors and nurses should be based entirely on what is best for them and their families rather than for SA’ and ‘Governments have no right to implement policies that restrict the migration of HPs to and from countries’), which increased the alpha reliability for the Attitudes scale to 0.66.

### Procedure

The medical schools and nursing school were contacted about the study to request their permission. After receiving their approval for the study, the researchers liaised with relevant course coordinators to arrange times for the researchers to meet the students and disseminate the questionnaires. All the medical and the nursing schools were visited by a researcher except for one where the researchers arranged with a course coordinator to administer the questionnaires. Prior to completing the questionnaires, each student was informed about the study and the voluntary nature of their participation. All signed consent forms.

### Analysing

The data from the questionnaires were entered into the Statistical Package for the Social Sciences (SPSS) version 18. The data were analysed using various descriptive and inferential statistical tests. Descriptive statistics including frequency counts, cross-tabulations, means and standard deviations were used, followed by inferential statistics including T-tests, Chi-Squares and ANOVAs. This enabled the statistically significant data to be identified with the descriptive data, lending to its interpretation. Only significant results were reported unless specific results were unexpected. Scales were computed for all attitude items to form an Attitude Scale and the belief items were computed to form a Belief Scale.

## Results

Most respondents were 19 to 24 years of age (73%, *n* = 214), SA citizens (91%, *n* = 199) single (92%, *n* = 272), had no dependents (*n* = 203) and lived in urban areas (57%, *n* = 164). More females (*n* = 58%, 172) than males (42%, *n* = 124) participated in the study; the largest number were self-reported Black people (45%, *n* =106), followed by White people (44%, *n* =103).

### Intentions to emigrate after completing their community service

Of the respondents, 77.4% (*n* = 185) reported that they had given ‘some’ (53%, *n* = 127) or ‘a great deal’ of consideration (25%, *n* = 58) to moving abroad. Over a quarter of the respondents intended to specialise overseas rather than in SA (28%, *n* = 82), although 35% (*n* = 103) reported intending to specialise in SA. The most popular destinations identified by respondents were countries in North America (19%, *n* = 56), the UK (18%, *n* = 53), and other countries within the European Union (9%, *n* = 27). About half the sample (51%, *n* =122) reported being ‘likely’ or ‘very likely’ to live abroad for longer than two years, whilst 62% (*n* = 144) thought they would live abroad for two years or less. There was no significant relationship between respondents’ intentions to work or specialise abroad and their gender, citizenship or the encouragement given by their educational institution to work and train abroad.

Table 1 illustrates the frequencies and percentages of medical and nursing students and the likelihood of their moving abroad within two and five years after graduating. The results showed a significant difference in the proportion of medical respondents and nurses who would like to move abroad within two years of graduating, *X*^2^ (6, *n* = 213) =29.47, *p* < 0.01. A larger proportion of nursing students (21%, *n* = 5) compared to medical students (18%, *n* = 33) reported being ‘likely’ to leave SA within two years of graduating, whilst 29% (*n* = 7) of nursing students compared to 9% (*n* = 17) of medical students indicated they were ‘very likely’ to leave the country within two years of graduating. Approximately two thirds of the sample (63%, *n* = 145) indicated that they were not likely to leave the country for at least two years after graduating, whilst 28% (*n* = 64) thought they would probably have left SA by that time. This increases dramatically, however, when respondents were asked whether they were likely to leave SA within five years. Almost half of the respondents (46%, *n* = 137) indicated that they were ‘likely’ or ‘very likely’ to leave SA within five years of graduation. Only 55 respondents (19%) were certain that they would remain in the country for at least five years after graduating.

**TABLE 1:** Emigration potential of medical and nursing respondents within two and five years after graduation (*n* = 298).

Profession	Likelihood of moving from SA within two years after graduation†								Likelihood of moving from SA within five years after graduation†							
		
	Unsure		Not likely		Likely		Very likely		Unsure		Not likely		Likely		Very likely	
Medical students	16	7.7	139	67.2	35	16.9	17	8.2	32	15.5	53	25.7	75	36.4	46	22.3
Nursing students	6	25	6	25	5	20.8	7	29.2	6	25	2	8.3	8	33.3	8	33.3

† Missing data not included.

The amount of planning and preparation undertaken to move abroad significantly varied according to respondents’ intentions to work overseas, *χ* ^2^ (20, *n* = 228) = 159, *p* < .01. The majority of respondents (79%, *n* = 84) who intended to work overseas after their community service had engaged in some form of planning and preparation to move abroad. Significant differences in the amount of planning and preparation undertaken were found between respondents who were certain they would emigrate within two years after graduation and those who were less certain, *χ* ^2^ (12, *n* = 226) = 45.54, *p* < .01 and between those who were certain they would emigrate within five years of graduating and those who were less certain, *χ* ^2^ (12, *n* = 225) = 39.26, *p* < .01. Most respondents who indicated that they would emigrate, either within two years (59.4%, *n* = 38) or five years of graduating (70%, *n* = 96), had already started planning for their move.

### Respondents’ attitudes and beliefs about emigration

Almost all the respondents reported that their medical or nursing school were not encouraging them to work (92.7%, *n* = 268) or train abroad (88.2%, *n* = 255). The mean score for the Belief Scale (11 items) was 34.72, with a standard deviation of 7.42. The mean score for the Attitude Scale (10 items) was 26.96, with a standard deviation of 4.48. Correlation analysis between the total number of scores for the Attitude and Belief Scales shows that these scales were not significantly related to one another (*r* = .06, *p* = .59). Respondents generally had positive beliefs about the prospects and benefits of emigrating from SA. These are illustrated in Table 2. The beliefs about emigration which garnered the highest consensus amongst respondents related to aspects of their medical careers rather than their quality of life abroad. This included the belief that there are better prospects for career advancement abroad (62%, *n* = 142), that they will get higher salaries if they work abroad (87%, *n* = 202), and that they would be exposed to fewer occupational risks if they worked abroad (66%, *n* = 155) than would be the case in SA.

**TABLE 2:** Positive beliefs about emigrating from SA reported by sample respondents (*n* = 298).

Belief statement†	Agreeing with statement		Uncertain	
		
	*n*	%	*n*	%
I will be able to find the job that I want abroad	175	58.7	43	18.5
There are better prospects for career advancement abroad	142	61.8	48	20.9
I will get a higher salary working abroad	202	85.6	18	7.6
The Occupational Specific Dispensation will not ensure that doctors and nurses remain in SA	57	24.4	48	20.5
I will experience better job satisfaction if I work abroad than if I stay in SA	109	46.5	46	19.7
I will be exposed to fewer occupational risks abroad	155	66.2	37	15.8
Nurses and doctors who have worked abroad are highly recognised in their profession	116	49.4	48	20.4
It is much safer for me and my family to live abroad	132	56.4	44	18.8
My family and I will have a better quality of life if we live abroad than if we stay in SA	121	51.3	46	19.5
I will be happier working in my profession abroad than if I remained in SA	53	31.1	58	24.7
I will have the same or a higher standard of living abroad than in SA	127	54	46	19.6

† Missing data not included.

Respondents who intended to embark on further training or specialisation abroad held significantly more positive beliefs about moving abroad (*M* = 37.08, s.d. = 7.40) than those who did not intend to train abroad (*M* = 32, s.d. = 8.45), or who were unsure about this possibility (*M* = 34.61, s.d. = 5.73), *F*(2, 79) = 3.19, *p* < .05. There was no significant difference in emigration beliefs between those respondents who wanted to, or did not want to work overseas after their community service (*p* = .93), or between different destination countries identified by respondents (*p* = .12).

Respondents tended to hold negative attitudes about emigration, tending to be in favour of HPs remaining in SA to work (Table 3). The majority of respondents did not feel that the South African government was to blame for the emigration of HPs based on poor working conditions (79%, *n* = 193) or low salaries (83%, *n* = 203) and believed that the government has a right and responsibility to introduce measures to reduce the emigration of South African health workers (79%, *n* = 193). Attitudes towards emigration were not found to be significantly associated with intentions to work abroad (*p* = .82), intentions to train abroad (*p* = .07), or respondents’ most likely destination country (*p* = .29).

**TABLE 3:** Positive and negative attitudes towards emigration reported by sample respondents (*n* = 298).

Attitude statement	Belief statement†	Agreement with statement		Disagreement with statement	
			
		*n*	%	*n*	%
Positive attitudes towards emigration	Doctors and nurses who remain in SA for their entire careers are not ambitious or motivated to advance in their profession	230	84.9	41	15.2
Negative attitudes towards emigration	It is wrong for nurses and doctors to leave SA to work abroad	215	78.8	58	21.3
	Doctors and nurses have a responsibility to work in the country that helped to train them	164	61	105	39.1
	The emigration of health workers is responsible for SA's poor health care system	161	59.4	110	40.6
	I am supportive of Government action and policy that encourages the return of health workers from abroad	99	37.1	168	62.9
	Governments are responsible for the health needs of their population, even if this means limiting the freedom of HPs to emigrate abroad	190	70.3	80	29.6
	Doctors and nurses who emigrate are robbing the country of much needed skills and expertise	165	60.9	106	39.2
	Working abroad will give HPs experience and skills they will not acquire in SA	109	40.3	161	59.6
	Health workers leave the country because the government does not pay them enough	42	15.8	224	84.2
	Emigration decisions should be based entirely on what is best for the person and their family	78	29	191	71
	Governments have no right to implement policies to restrict emigration	54	20.1	213	79.5
	It is government's responsibility to take steps to limit emigration to make sure the health needs of the country are met	190	70.3	80	29.6
	Governments’ failure to improve working conditions in the public sector is causing health workers to emigrate	17	6.3	252	93.4

† Missing data not included.

SA, South Africa; HP, healthcare practitioners.

### Factors associated with intentions to emigrate from SA

Table 4 presents a list of the push and pull factors that were identified to influence respondents’ decisions to move abroad. They are listed in order of importance. The push factors that exerted the greatest influence on respondents’ intentions to leave SA related to the working conditions and environments in which they would one day practice as doctors and nurses in SA. Working conditions in the public sector (53.7%, *n* = 123) were identified as the strongest push factor encouraging the medical and nursing respondents to leave the country, followed by the heavy workloads of nurses and doctors (49.1%, *n* = 95) and inadequate medicines, supplies and equipment (51.5%, *n* = 118). Of all the push factors related to respondents’ quality of life and social environment, crime and violence (41.6%, *n* = 94) was reported to play the greatest role in respondents’ plans to leave the country. The extent to which push factors influenced emigration plans did not vary according to the countries where respondents wanted to migrate to.

**TABLE 4a:** Push factors influencing medical and nursing students’ emigration decisions (*n* = 298).

Push factors†	Indicating it was a ‘great influence’ on their emigration decisions	
	
	*n*	%
Working conditions in the public sector	123	53.7
Inadequate medicine supplies and equipment	118	51.5
Heavy workloads for doctors and nurses	112	49.1
Risk of occupational exposure (e.g. to TB, HIV)	95	41.7
Crime and violence	94	41.6
Low salaries	77	33.5
Political instability in SA	68	29.7
Deterioration of quality education	67	29.6
Current economic conditions in SA	62	27.2
Poor quality of life	57	25.2
Uncertainty about SA's future	54	23.7
Working conditions in private sector	54	23.7
Lack of opportunities for career advancement	48	21
Cost of living in SA	29	12.8
I won't find a job in SA	17	7.5

Note: Listed in order of frequency counts

† Missing data not included.

SA, South Africa

**TABLE 4b:** Pull factors influencing medical and nursing students’ emigration decisions (*n* = 298).

Pull factors†	Indicating it was a ‘great influence’ on their emigration decisions	
	
	*n*	%
Higher salaries in destination country	110	47.8
Quality and variety of speciality training offered	107	47.3
Prospects for professionals’ advancement	99	43.6
Greater personal safety abroad	90	40
Availability of good jobs abroad	90	39.8
Potential to earn more money abroad to put towards a private practice in SA	86	37.9
The quality of life abroad	85	37.9
The standard of living abroad	85	37.8
The economic situation of destination country	83	37.4
Better opportunities for family abroad	76	33.8
Stability of the national government in destination country	73	32.6
Transferability of qualification	67	29.8
Ability to repay study loan or other financial commitments sooner	65	28.8
Similarities in health care training between SA and destination country	25	11.1

Note: Listed in order of frequency counts

† Missing data not included.

SA, South Africa

The strongest pull factors attracting respondents to emigrate abroad included the opportunity to earn higher salaries (48%. *n* = 110), the quality and variety of specialist training offered (47.3%. *n* = 107), prospects for professional advancement (44%. *n* = 99), the availability of good jobs (40%, *n* = 90) as well as greater personal safety abroad (40%, *n* = 90).

Table 5 provides the frequency counts and percentages of respondents that identified certain obstacles as either ‘somewhat of a barrier’ or a ‘great barrier’. The costs associated with emigrating (e.g. for visas, examination fees and plane tickets) (40%, *n* = 107), having dependents in SA (40%, *n* = 105), obligatory work commitments respondents would be involved in after their community service (38%, *n* = 101) and ease of adjustment to the healthcare system in their destination countries (34%, *n* = 90) were the most important barriers to emigration. A smaller proportion of respondents indicated that government policies in SA (24%, *n* = 64) and in their destination country (25%, *n* = 67) and qualifying for visas in their destination country (6%, *n* = 23) were substantial barriers deterring them from moving abroad.

**TABLE 5:** Perceived barriers preventing respondents from emigrating from SA.

Most important barriers†	Indicating obstacle was ‘somewhat of a barrier’ or a ‘great barrier’ to moving abroad	
	
	*n*	%
Associated costs	107	40.2
Dependants	105	38.9
Obligatory work commitments after community service	101	38.3
Adjusting to MLD health care system‡	90	34.1
Certification	88	33.2
Government policies in MLD	67	25.4
Government policies in SA	64	24.4
Adjusting to MLD culture	47	17.9
Qualifying for visas	23	6.1

† Missing data not included

‡ This item referred to the perceived ease of adjustment to working within the healthcare system of their MLD, in terms of the language, medical and nursing training background and burden of disease they would encounter in their MLD.

## Discussion

Irrespective of their destination countries, about a quarter of the respondents (28%, *n* = 64) would probably leave SA within two years of graduating, and as many as 59% (*n* = 137) would leave within five years of graduating. Other researchers have reported that as many as 65% of health professional students in sub-Saharan Africa intend to emigrate within five years of graduating.^6^ This study did not elicit any data about their reasons for delaying their emigration for five years after graduation, and therefore only tentative explanations for this waiting period can be proposed. Respondents may be in favour of waiting a few years before moving abroad in order to determine whether emigrating abroad is the right move to make, or perhaps in order to use this time to gain work experience, thereby improving their prospects for employment abroad. Alternatively, respondents may be in favour of first specialising (a four year programme for most medical specialisations) in SA before leaving the country. Given that the costs associated with emigrating was the most common barrier reported by 40% (*n* = 107), it is possible that the purpose for this waiting period may also involve saving money to pay for emigration costs. Irrespective of when they would leave, almost two thirds of the sample (62%, *n* = 144) anticipated moving abroad for two years or less at some point in their careers, whilst about half the sample (51%, *n* = 122) contemplated a move of two years or more abroad. This does suggest that many students considered the possibility of permanently remaining abroad. The majority of the respondents who intended to work abroad after graduation had started some form of planning and preparation for such moves. This finding suggests that most of the respondents who wanted to move abroad were in fact seriously interested in and thinking about doing so.

The results also showed that there were significant differences in the proportion of medical respondents and nursing respondents who would like to move abroad after two years of graduating. Although far fewer nursing students participated in the study compared to medical students, the findings suggest that a higher proportion of nursing respondents than medical respondents would like to emigrate within two years after graduating. Other researchers have also noted the high emigration potential amongst young nurses in SA, arguing that young people are increasingly more likely to choose the nursing field as a career, even if it is not their preferred choice, because of the emigration opportunities and better career prospects it offers them.^15^

### Respondents’ attitudes and beliefs about emigration

Positive beliefs about living and working abroad were more likely amongst those intending to avail themselves of further training and specialisation opportunities abroad. The most prevalent positive beliefs about emigration related to aspects of the job, such as fewer occupational risks, higher salaries and better career advancement opportunities. Very few felt that HPs emigrated because HPs are underpaid in SA, suggesting that salaries may be not be a significant factor in emigration decisions. This is supported by research which showed how the introduction of the OSD^1^ in SA has significantly increased salaries for nurses and doctors in relation to their international counterparts.^16^

In terms of the respondents’ attitudes to emigration, the findings showed that in general most of the respondents were supportive of HPs remaining in SA for the duration of their careers and government actions and strategies to curb the emigration of HPs. These negative attitudes towards emigration may be influenced by the stance of their educational institutions on the emigration of HPs, given that the majority of the sample reported that their educational institution was not encouraging them to work or train abroad. The general attitude against emigration does contradict the high prevalence of emigration intentions reported by the sample. Moreover, the lack of association between respondents’ attitudes and beliefs about emigration suggests that some respondents could hold these relatively strong attitudes against emigration alongside positive beliefs about the working conditions and job prospects of working abroad. It seems that holding onto values and ideals that disapprove of HPs leaving SA is not a guarantee that they will not want to work abroad.

### Factors associated with intentions to emigrate

Perceived low salaries of doctors and nurses was an important push factor for only a third of the sample (33.5%, *n* = 77) in comparison to the various push factors in the working environment that were strongly influencing between 41% and 53% of the sample to leave the country. These findings suggest that it is due to poor working conditions, including occupational hazards related to, *inter alia*, HIV and TB, which is the biggest factor influencing prospective doctors and nurses in the health sector to migrate. Factors related to crime and personal safety was found to be the most salient personal factor, unrelated to work, that motivated them to emigrate abroad. Similar findings have been found by other researchers. Previous research has found that doctors were significantly more likely than nurses to migrate for safety reasons^11^, whilst a SA study^17^ showed that 61% of HPs would like to leave SA because of safety and security issues.

Regarding the barriers that may hinder respondents from leaving the country to work or train abroad, the foremost barriers identified by respondents were the costs associated with emigrating such as the air fares, examination fees to acquire professional registration abroad and applications for visas and work permits. Furthermore, the high cost of emigrating may partially explain why most respondents intended to emigrate within two to five years of graduating, giving them time to save money to pay for the costs associated with moving abroad. Secondly, having dependents might deter some respondents from leaving the country, whether these be children or older family members. Adjustment to the culture of the healthcare system in the respondents’ destination countries was another prevalent barrier that emerged from the findings. This potential barrier may contribute to explaining why HPs prefer to emigrate to English speaking countries such as Canada, North America, Australia, New Zealand and the UK and also to other EU countries, as the results of this study show and other studies confirm.^1, 3^

Having to fulfil obligatory work commitments after their community service was another salient barrier deterring many of the respondents from moving abroad and it was surprising to find that more than a third of the sample had obligatory work commitments to fulfil. Our results show that working abroad to earn money to repay financial commitments sooner than would be possible if they remained in SA, played an important role in approximately a third of the respondents’ emigration plans. Given that 42.9% (*n* = 118) of the respondents indicated that they had received government bursaries and scholarships, this may culminate in huge losses for the SA government.

The findings also showed that qualifying for visas and government policies in SA and their most likely destination were least likely to be perceived as barriers hindering respondents from working abroad, compared with the other identified barriers. This suggests that respondents may be unaware of some countries’ stringent immigration policies, also regarding the recruitment and employment of foreign HPs. Respondents were in the early stages of planning and preparing for moving abroad and therefore they may not have been familiar with their destination countries’ policies regarding immigration and the employment of foreign doctors and nurses, or they may merely have started the process of applying for visas and work permits.

### Limitations of the study

The study is limited by its use of a cross-sectional design that entailed the gathering of data at only one point in time from medical and nursing students in three medical schools and one nursing school. The use of purposive sampling to sample only medical and nursing students in their prefinal and final years does not allow us to generalise these findings to medical and nursing students in earlier years of study, or other types of health profession students, nor to other provinces in SA. The generalisation of these findings to other medical and nursing schools is further limited by the fact that only a small number of medical and nursing schools participated in the study.

### Recommendations

It is widely believed that the perceived low salaries of HPs are a primary reason for HPs wanting to leave the country. However, these findings suggest that it may be that the salaries of nurses and doctors are only considered ‘low’ in relation to the challenging and under-resourced working environments they are forced to endure in the public sector.

The most important factors influencing respondents to move abroad was a better job with better career prospects and training opportunities. Lifestyle factors such as a higher standard of living, quality of life and better opportunities in their destination country were found to exert less influence on their decisions, suggesting that perhaps the perceived gain in lifestyle-related factors when living abroad was considered to be far smaller than the gains to be made in work-related factors.

The predominance of work-related factors over lifestyle-related factors in the students’ emigration decisions may relate to the time period they find themselves in, where few have families and/or dependents and work-related concerns are therefore a higher priority. To this end, it would be important for longitudinal research studies to be conducted in order to contribute towards an understanding of dominant push and pull factors across health professionals in different age groups and career trajectories in order to develop retention strategies and incentives tailored to health professionals’ needs and circumstances.

Furthermore, the findings revealed that intentions to emigrate were relatively common amongst both the medical and nursing students in our samples. The fact that countries within the EU and the UK in particular remain a popular destination for student respondents indicates that inflows of South African HPs into the EU will probably continue into the future even with the introduction of the Professional and Linguistics Assessments Board (PLAB) exams and stricter policies regarding the recruitment and employment of foreign HPs. It appeared that many student respondents would wait for an initial period after graduating or completing their community service before deciding whether to leave the country, during which time most of them would either specialise, work as junior doctors and nurses, or fulfil obligatory work commitments associated with their financial assistance. This highlights a critical early period in the careers of junior doctors and nurses during which many are prone to want to emigrate. Given the large investment by the government into the training of nurses and doctors in the country, the fact that a majority of doctors and nurses would emigrate early in their careers is of concern and represents a potential major loss of investment for the country.

There is much concern about the loss on investment incurred by host countries when medical students move abroad after completion of their studies, particularly those whose studies have been subsidised by governments in developing nations.^18, 19^ The importance of selecting the right candidates for financial assistance to protect the return of an investment spent on training health workers is underscored by the fact that over half the sample receiving financial assistance did so in the form of government bursaries and scholarships. Furthermore, the opportunity to repay financial commitments was a strong motivator influencing more than a third of the sample to emigrate. As the findings show, there is a small contingent of medical and nursing students who are resolved to remain in the country, and who should be the target of government bursaries and scholarships to ensure a better return on investment in the training of HPs in the future. In addition, it is of utmost importance that doctors and nurses feel adequately engaged by the South African government with regard to their complaints about working conditions, low salaries and heavy workloads during the critical early period in their careers. This carries with it implications for efforts to improve the community service experience of nurses and doctors to ensure they come through their year of community service with a desire to remain in the country.

## Conclusion

Although the majority of respondents were in favour of moving abroad temporarily and for a short period of time, a slightly lower than equal proportion were intending to leave SA permanently or were unsure as to how long they would be gone. The introduction of time-limited placements or other structured programmes that provide these HPs with the experience of working and living abroad they desire, but also contribute to ensuring they return to SA after their stay abroad may be an effective strategy to prevent the loss of HPs who go abroad for temporary or indefinite periods of time. In this regard, the positive benefits of temporary migration for South African HPs must not be overlooked, as these periods can provide them with enhanced skills in patient care and evidence-based medicine, training opportunities and experience with advanced equipment. Recruitment companies that have experience in assisting South African HPs abroad to return home to employment opportunities in the public service may provide valuable assistance in this area.

## Footnotes

1 The development and implementation of the OSD for HRH was based on the recognition that the improvement in the conditions of service and remuneration for health professionals constituted an urgent priority. Announced in 2007, the OSD resulted in all HRH in the public service being re-graded according to their qualifications and years of experience, remunerated accordingly, with the programme implemented over five years
